# Dual-Wavelength Forward-Enhanced Directional Scattering and Second Harmonic Enhancement in Open-Hole Silicon Nanoblock

**DOI:** 10.3390/nano12234259

**Published:** 2022-11-30

**Authors:** Xinghua Wang, Yunbao Zheng, Min Ouyang, Haihua Fan, Qiaofeng Dai, Haiying Liu

**Affiliations:** 1 Guangdong Provincial Key Laboratory of Nanophotonic Functional Materials and Devices, School for Information and Optoelectronic Science and Engineering, South China Normal University, Guangzhou 510006, China; 2 School of Optoelectronic Engineering, Guangdong Polytechnic Normal University, Guangzhou 510665, China

**Keywords:** open-hole silicon nanoblock, electromagnetic multipole resonance, directional forward-scattering enhancement, second harmonic generation enhancement

## Abstract

Nanostructures with appropriate sizes can limit light-matter interaction and support electromagnetic multipole resonance. The interaction between light and nanostructures is intimately related to manipulating the direction of scattered light in the far field as well as the electromagnetic field in the near field. In this paper, we demonstrate dual-wavelength directional forward-scattering enhancement in an individual open-hole silicon nanoblock (OH-SiNB) and simultaneously achieve bulk and surface electromagnetic field localization. The second harmonic generation is enhanced using electromagnetic field localization on the square hole surface. Numerical simulations reveal that the resonance modes, at *λ*_1_ = 800 nm and *λ*_2_ = 1190 nm, approximately satisfy the Kerker condition. In the near field, the magnetic dipole modes at dual wavelength all satisfy the boundary condition that the normal component of the electric displacement is continuous on the square holes surface, thus obtaining the surface electromagnetic field localization. Moreover, highly efficient second harmonic generation can be achieved at dual wavelengths using the surface electromagnetic field localization and the increased surface area of the square holes. Our results provide a new strategy for the integration of nanoantennas and nonlinear optoelectronic devices in optical chips.

## 1. Introduction

Silicon is one of the most promising materials for developing advanced optoelectronic devices due to the significant advantages of low cost and mass production [1,2]. Silicon-based optoelectronic devices are critical components in the fields of all-optical switching, generation of entangled photons, optical waveguides, sensing and detection [3,4,5,6,7,8]. With the rapid development of nanofabrication technology, researchers have discovered that unique optical responses and interesting optical phenomena are generated by changing the shape and size of silicon nanostructures [9,10,11,12,13]. These findings have practical applications in the area of magneto-optics generation and the design of nanoantenna devices [14,15]. The main reason for the wide interest in silicon is its relatively high refractive index, high thermal resistance, low absorption and optical losses in the visible to near-infrared region compared to metallic materials [16,17]. Because of the high refractive index and low absorption properties of silicon, nanostructures can support electromagnetic multipole resonances when light interacts with them. Electric dipole (ED) resonance, magnetic dipole (MD) resonance and other higher-order resonances, such as electric quadrupole (EQ) and magnetic quadrupole (MQ), can be simultaneously observed in a single nanostructure [13,18,19]. The interference between these multipole resonances is of great interest in the design of directional nanoantennas. Kerker et al. found that the ED and MD resonances of equal amplitude can achieve zero backscattering when the phase difference is π [20]. In addition, the generalized Kerker condition for the interaction of dipole modes with higher-order modes, such as quadrupole modes, can also lead to directional far-field radiation [21,22]. Therefore, the interference between the resonance modes injects new dynamics into the radiative manipulation of light in the far field. In recent years, a remarkable amount of progress has been made in the manipulation of optical fields in the far field. For instance, narrow-band, single-wavelength directional scattering of gold-silicon spherical heterodimers in the infrared band was investigated [23]. Furthermore, the directional forward scattering of broadband in the visible light band by a single silicon nanocube as well as forward-scattering enhancement and backward-scattering suppression by a single silicon nanodisk or Si/SiO_2_ core-shell structure have also been successfully performed [11,12,24]. However, studies on the multi-wavelength enhanced directional forward scattering of silicon nanostructures in the infrared band have been rarely reported.

Electromagnetic multipole resonance can also manipulate near-field enhancement [25,26,27]. The localization of strong electromagnetic fields generated by electromagnetic multipole resonance creates conditions in which nanostructures can enhance nonlinear optical responses [28,29]. Due to the strong localized electromagnetic field properties of silicon nanostructures, they have unique manipulation capabilities in nonlinear regions [30]. In the near-infrared band, the researchers have effectively enhanced the third harmonic generation in silicon nanostructures utilizing the electromagnetic field localization generated by electromagnetic multipole resonance [31]. However, although silicon has a strong third-order nonlinear response, it is a centrosymmetric crystal that lacks the bulk second-order nonlinear optical responses. Thus, the second-order nonlinear signal is very weak relative to the third-order nonlinear signal. The broken centrosymmetry is allowed at surface or interface, making it possible to achieve silicon-based second-order nonlinear responses [32,33]. Unfortunately, most of the electromagnetic fields generated by electromagnetic multipole resonances are bulk-localized and the ineffective light-matter interaction in the body cannot achieve effective second-harmonic generation (SHG) enhancement [34]. Therefore, it is necessary to design appropriate nanostructures to achieve SHG enhancement in centrosymmetric crystals.

In this paper, we propose directional forward-scattering enhancement that can be realized by interference between electromagnetic multipole resonance modes in the OH-SiNB through numerical simulation. At *λ*_1_ = 800 nm, the interaction of the MD_1_ and TED modes with the EQ mode results in directional forward-scattering enhancement due to the satisfaction of the generalized Kerker condition, and the presence of the EQ mode produces a small backscatter. At *λ*_2_ = 1190 nm, a conventional MD mode and the TED mode interference that approximately meets the first Kerker condition result in near-zero backscattering. Since both the MD_1_ mode and the MD mode generated by the electromagnetic multipole resonance in the near field satisfy the continuity condition of the normal component of the electric displacement, a localized electromagnetic field on the surface of the square holes of the silicon nanoblock is obtained. The electromagnetic field localization on the surface of the square holes and the increased surface area of the square holes effectively realizes the SHG enhancement at dual-wavelength in the OH-SiNB.

## 2. Materials and Methods

**Figure 1:**
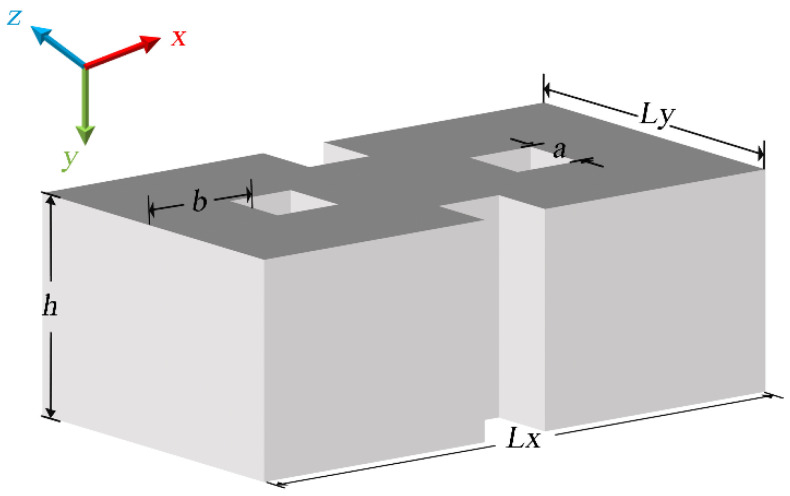
Schematic diagram of the structure of the OH-SiNB.

**Figure 2:**
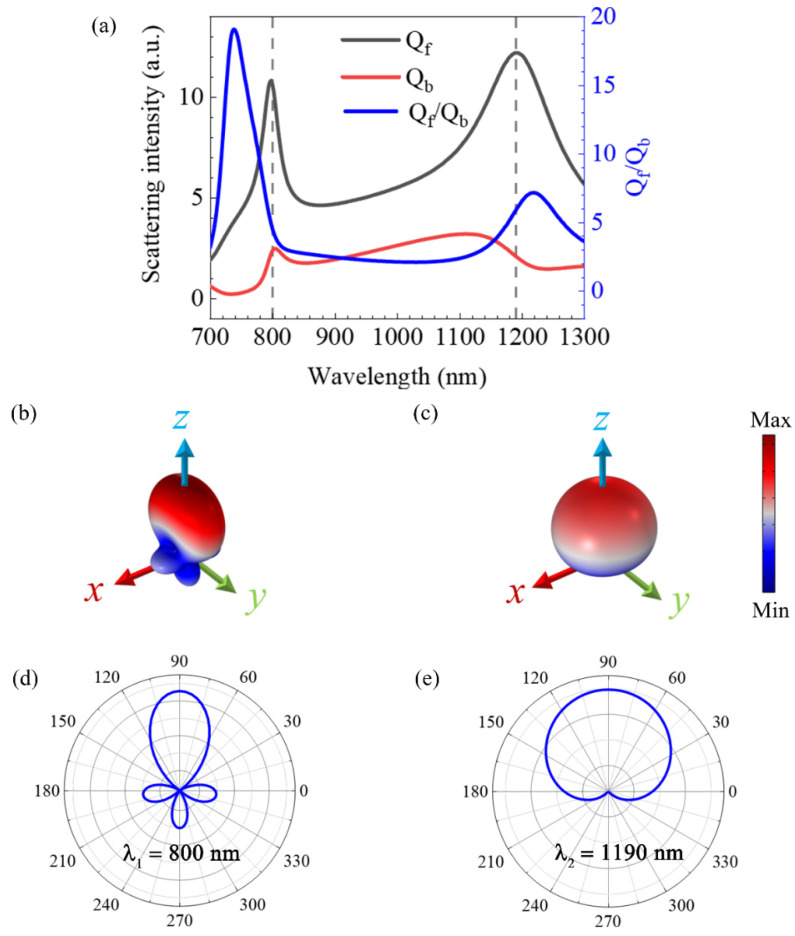
(**a**) Power spectrum of forward scattering (*Q_f_*), power spectrum of backward scattering (*Q_b_*) and ratio of intensity of forward and backward scattering (*Q_f_ ⁄Q_b_*). Far-field radiation properties for (**b**) *λ*_1_ = 800 nm and (**c**) *λ*_2_ = 1190 nm. Angular plots of far-field radiation (*xoz* plane) at (**d**) *λ*_1_ = 800 nm and (**e**) *λ*_2_ = 1190 nm.

We calculated the various components of the electromagnetic multipole resonance theory in the OH-SiNB and studied its scattering properties in free space (*ε_d_* = 1) using the commercial software COMSOL Multiphysics 6.0 (https://cn.comsol.com/). The dielectric constants of the crystalline silicon were taken from Palik [35]. Figure 1 shows the schematic diagram of the designed nanostructure. The geometric parameters of the OH-SiNB were different in three dimensions with *L_x_* = 420 nm, *L_y_* = 240 nm and *h* = 185 nm. The length of the square holes was *a* = 25 nm. The distance from the left boundary of the square hole to the left boundary of the silicon nanoblock was *b* = 122.5 nm, and the two square holes in the middle were symmetrically distributed around the *z*-axis. The wave vector of the excitation plane wave was along the *z*-direction and the polarization was along the *x*-axis. The scattered power spectra of the forward and backward hemispheres in free space are shown in Figure 2, where the forward direction is along the incident light and the backward direction is the opposite of this. We neglected the presence of the substrate since it had no effect on the scattering spectral properties of a given nanostructure and only redshifted its resonant wavelengths [36].

We used the Cartesian multipole decomposition theory to analyze the electromagnetic multipole resonance modes in the OH-SiNB [18]. The multipole moment comes from the polarization ***P***(**r**) = *ε_d_* (*ε_p_* − *ε*_0_) ***E***(**r**) generated by the incident light, where ***E***(**r**) is the total electric fields of the scatterer. *ε*_0_, *ε_p_*, *ε_d_* are the free-space permittivity, the relative permittivity of the nanoblock and the relative permittivity of the surrounding medium, respectively. The multipole is located at the origin of the Cartesian coordinate system according to the center of mass of the scatterer.

The ED moment can be expressed as:

(1) $$\vec{P}=\int_{V}^{\;}P\left(r^{\prime }\right)dr^{\prime }$$

where *V* is the volume of the scatterer and ***r’*** is the vector radius of the internal volume element of the scatterer.

The toroidal dipole (TD) is a toroidal multipole characterized by the vortex distribution of the magnetic dipole and TD polar moment is a higher order term of electric dipole. The TD moment can be expressed as follows:

(2) $$\vec{T}=\frac{i\omega }{10}\int_{V}^{\;}\left\{2{r^{\prime }}^{2}P\left(r^{\prime }\right)-\left[r^{\prime }\cdot P\left(r^{\prime }\right)\right]r^{\prime }\right\}dr^{\prime }$$

The MD moment of the scatterer can be described as:

(3) $$\vec{m}=-\frac{i\omega }{2}\int_{V}^{\;}\left\{{r^{\prime }}^{2}\times P\left(r^{\prime }\right)\right\}dr^{\prime }$$

The EQ, MQ tensor can be written as:

(4) $$\hat{Q}=3\int_{V}^{\;}\left\{r^{\prime }P\left(r^{\prime }\right)+P\left(r^{\prime }\right)r^{\prime }-\frac{2}{3}\left[r^{\prime }P\left(r^{\prime }\right)\hat{U}\right]\right\}dr^{\prime }$$

(5) $$\hat{M}=\frac{\omega }{3i}\int_{V}^{\;}\left\{\left[r^{\prime }P\left(r^{\prime }\right)+P\left(r^{\prime }\right)\right]r^{\prime }+r^{\prime }\left[r^{\prime }\times P\left(r^{\prime }\right)\right]\right\}dr^{\prime }$$

The total electric dipole (TED) moment is written as:

(6) $$\vec{D}=\vec{P}+\frac{ik_{0}}{c}\varepsilon_{d}\vec{T}$$

Finally, the total far-field scattering power *P_sca_* for a single OHSiNB is given considering the above multipole moments.

(7) $$P_{sca}\;≅\;\frac{k_{0}^{4}}{12\pi \varepsilon_{0}^{2}v_{d}\mu_{0}}{\left|p+\frac{ik_{d}}{v_{d}}T\right|}^{2}+\frac{k_{0}^{4}\varepsilon_{d}}{12\pi \varepsilon_{0}^{2}v_{d}}{\left|m\right|}^{2}\;\;\;\;\;\;\;\;\;\;\;\;\;\;\;\;+\frac{k_{0}^{6}\varepsilon_{d}}{1440\pi \varepsilon_{0}^{2}v_{d}\mu_{0}}\sum_{\alpha \beta }{\left|Q_{\alpha \beta }\right|}^{2}+\frac{k_{0}^{6}\varepsilon_{d}}{160\pi \varepsilon_{0}^{2}v_{d}}\sum_{\alpha \beta }{\left|M_{\alpha \beta }\right|}^{2}$$

In the above equation, α=x,y,z, β=x,y,z and $\mu_{0}$ is the vacuum permeability. *v_d_* is the speed of light propagation in the medium. *k*_0_ and *k_d_* represent the wave numbers in the vacuum and in the same surrounding medium, respectively.

## 3. Results and Discussion

### 3.1. Dual-Wavelength Forward-Enhanced Directional Scattering Effect

The scattered power spectra of the OH-SiNB excited by the linearly polarized plane wave are shown in Figure 2. The forward-scattering spectra (black curve) and backward-scattering spectra (red curve) are shown in Figure 2a, along with the ratio of forward to backward (blue curve) calculated from the forward- and backward-scattering spectra. In the near infrared band, the forward scattering of the OH-SiNB dominated, and there are two distinct maxima in the forward scattering curve, which are the wavelengths *λ*_1_ = 800 nm and *λ*_2_ = 1190 nm, corresponding to the vertical gray dashed lines. This behavior suggests that the OH-SiNB can achieve directional forward-scattering enhancement in the near-infrared band of our study. The forward-to-backward ratio at the peak *λ*_1_ is lower than that at *λ*_2_, which indicates that there is relatively strong backward-direction-scattered light at *λ*_1_, as shown in Figure 2a. It is worth noting that the peaks of *Q_f_/Q_b_* cannot coincide with those of *Q_f_*. The main reasons for this are that the electric and magnetic resonances are usually spectrally separated from each other and that the phase difference of the resonance modes cannot be strictly equal to π, as well as the electric and magnetic resonance modes not being fully coupled. As a result, the forward-directional scattering *Q_f_* peak cannot be generated at the *Q_f_/Q_b_* peak [37]. We can observe that in the far-field emissions pattern at *λ*_1_ given in Figure 2b, the scattered light is clearly concentrated in the front hemisphere and there is also a certain amount of scattered light in the back hemisphere. The far-field radiation pattern at *λ*_2_ better satisfies the first Kerker condition and achieves near-zero backscattering. Additionally, we compared the two-dimensional far-field radiation distribution in the *xoz* plane of Figure 2d,e. The two-dimensional radiation distribution shows that although there is undesired backward-scattered light at *λ*_1_, the radiation-direction angle is smaller at *λ*_1_ compared to the angle at *λ*_2_.

**Figure 3:**
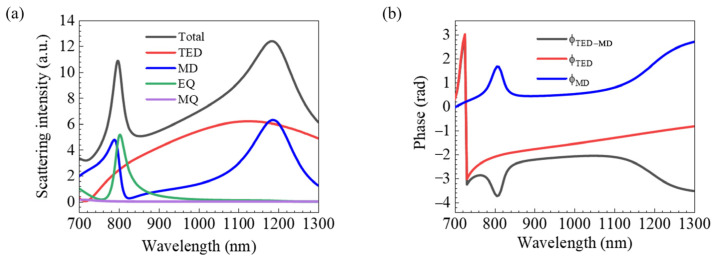
(**a**) Multipole decomposition scattering intensity, and (**b**) electric dipole and magnetic dipole phase and phase difference.

In order to reveal the difference between the far-field radiation modes and the internal resonance modes at the peak positions of the two forward-scattering maxima, we demonstrate the decomposition results of the electromagnetic multipole resonance of the OH-SiNB. There is good agreement between the sum of contributions from each multipole moment shown above (the black solid line in Figure 3a) and the spectrum dominated by forward scattering (the black solid line in Figure 2a). Hence, the higher-order multipole modes are negligible. The scattering intensity of each component in the electromagnetic multipole resonance decomposition corresponds to the four terms in Equation (7). The contribution of each electromagnetic multipole moment is significantly different to the two scattering resonance peaks as the electromagnetic multipole resonance unfolds. In Figure 3b, the phase difference between TED and MD can be found by using the real and imaginary parts of the dipole moment, and the phase difference is as follows:

(8) $$\Phi =\tan^{-1}\left(\frac{Im\left(D_{x}\right)}{Re\left(D_{x}\right)}\right)-\tan^{-1}\left(\frac{Im\left(m_{y}\right)}{Re\left(m_{y}\right)}\right)$$

The phase difference (*Φ_2_* ≈ 18.6°) at *λ*_2_ dominated by the overlap of TED and MD resonances with nearly equal amplitudes approximately satisfies the first Kerker condition, leading to enhanced directional forward scattering and near-zero backscattering along the *z*-direction [20]. However, while there are TED and MD resonances with amplitudes and moderate phase differences (*Φ*_1_ ≈ 24.3°) at *λ*_1_, there is also a strong EQ resonance. It is worth noting that the electric and magnetic dipole moments shown here interact with the electric quadrupole to satisfy the generalized Kerker condition. The interactions between the three modes provide a new property of multimode directional scattering. Although EQ produces a small amount of undesired backscattered light, it greatly improves the directionality of forward-scattered light. This optical scattering property with a small radiation-direction angle in the far field enhances the application potential of the structure in nanoantennas.

**Figure 4:**
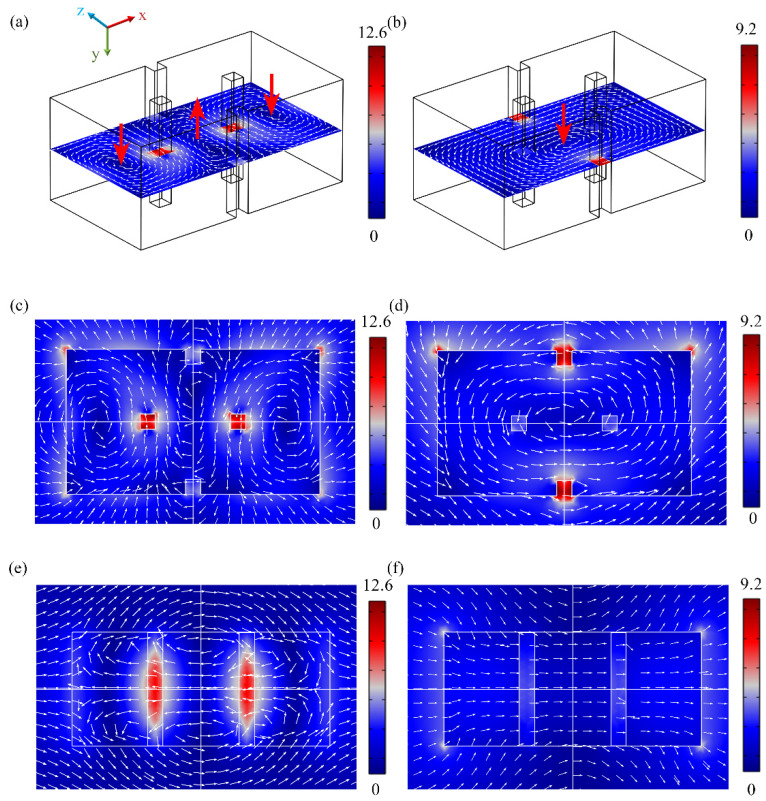
(**a**,**b**) Electromagnetic resonance modes in the OH-SiNB cavity at *λ*_1_ = 800 nm and *λ_2_* = 1190 nm, respectively; the red arrows indicate magnetic vector field. Electric-field (*|**E**|/|**E*****_0_**|) distribution in the (**c**) *xoz* plane and (**e**) *xoy* plane at *λ*_1_ = 800 nm. Electric-field distribution in the (**d**) *xoz* plane and (**f**) *xoy* plane at *λ_2_* = 1190 nm.

To further illustrate the internal resonance modes of the OH-SiNB at the two peaks, we plotted the near-field electric field distributions at *λ*_1_ and *λ*_2_*,* as shown in Figure 4. The optical resonant response in rectangular nanostructures is influenced by the size ratios of the different sides of the structure, which can decompose the electric and magnetic fields into the Fabry–Perot mode induced in a high-impedance cavity. The side boundary conditions of the rectangular dielectric structure determine the eigenfrequencies in the dielectric cavity, which can produce TM_nml_ modes in the *xoz* plane under the scattering conditions of the incident wave propagating along the *z*-axis [38]. Figure 4a shows the two magnetic dipole resonance modes influenced by the TM_301_ mode in the OH-SiNB cavity under scattering conditions at *λ*_1_ = 800 nm. The electric-field distribution in Figure 4c is the Fabry–Perot mode in the *x* and *y* directions, superimposed with the TED mode with electric vector polarization in the *x* direction and the MD mode in the *y* direction to form the novel magnetic dipole mode MD_1_. In addition, Figure 4a shows the MD_1_ mode consisting of two identical magnetic dipole moments $\vec{m}$. When the MD_1_ mode satisfies the condition of *p_x_* = 2*c*/*m_y_* (*c* is the speed of light), the first Kerker condition is satisfied. Therefore, the interaction of the MD_1_ and TED modes with the EQ mode satisfies the generalized Kerker condition and realizes the new feature of multimode forward-scattering enhancement [39]. A significantly enhanced electromagnetic field is located at the middle two holes shown in Figure 4e. The mode can be observed as TED mode through the surface current vector distribution distributed in this section and it is also influenced by the Fabry–Perot mode. The novel magnetic dipole mode influenced by the Fabry–Perot mode, as shown by the current vector distribution in the tangential plane Figure 4c, is two magnetic dipole moments with symmetrical distributions along the *y* direction and with a certain spatial distance *d*. Combined with the electromagnetic multipole resonance decomposition in Figure 3a, we further show that the enhancement of far-field directional forward-scattering enhancement results from the interference of the TED mode with the MD_1_ mode at *λ*_1_ = 800 nm. The EQ mode only produces a small amount of backscattered light in far-field. We observed that the electric-field distribution at *λ*_2_ = 1190 nm is dominated by an approximately equal amplitude in TED and MD in Figure 4d,f. The current vector in the tangent plane in Figure 4b,d indicates that this dipole is a typical conventional MD mode located at the center of the OH-SiNB. The dipole mode of Figure 4f is similar to that of Figure 4e. Hence, the TED and MD modes in the far-field radiation interfere constructively in the forward direction and destructively in the backward direction.

From the above analysis, it is clear that the unique patterns in the far field at the two peaks of the OH-SiNB support the enhancement of directional forward scattering. In near field, the local electric field at the position of the square holes shown in Figure 4 is significantly stronger than the local electric field in the silicon bulk. It is well known that there is almost no second-order optical nonlinearity contribution within the bulk of silicon [33], but OH-SiNB can be beneficial for the SHG enhancement of the centrosymmetric bulk materials. Two main reasons exist for the localization of strong surface electromagnetic fields in OH-SiNB. On the one hand, we make full use of the toroidal displacement currents formed by the novel magnetic dipole MD_1_ and the conventional magnetic dipole MD modes in the *xoz* plane. Therefore, a strong electric field localization on the surface occurs at *λ*_1_ = 800 nm and *λ*_2_ = 1190 nm in the square holes distributed along the *x*-axis and the *z*-axis, as shown in Figure 4a,c. This is mainly attributed to the continuous condition of the normal component of the electric displacement at the interface of square holes in the SiNB, i.e., the boundary condition *ε*_0_***E****_air_* = *ε_d_**E**_Silicon_* is satisfied [40]. On the other hand, the high refractive index property of silicon can better confine the electromagnetic field at the interface. Field enhancement can be impacted by the various contributions of the Fabry–Perot modes and the electromagnetic multipole resonance modes at dual wavelengths. Therefore, the square holes not only increase the surface area of the SiNB, but also effectively enhance the localization of the electromagnetic field on the surface of the square holes. The strong surface electromagnetic field locally promotes the interaction of light with atomic layers on the silicon surface, creating conditions for the enhancement of SHG.

### 3.2. Dual-Wavelength Second Harmonic Enhancement

The SHG of centrosymmetric materials is closely related to the local electromagnetic field on the surface [34]. Thus, we investigate its performance in SHG enhancement to further demonstrate the optical property of strong electromagnetic field localization on the surface of OH-SiNB. Since silicon is a centrosymmetric material, we describe the nonlinear optical response of the system using the nonlinear source polarization for the centrosymmetric medium and interface [34]. This response is the superposition of the surface dipole contribution and the bulk quadrupole contribution [33,41,42].

(9) $$P^{\left(2\omega \right)}\left(r\right)=P_{surface}^{\left(2\omega \right)}+P_{bulk}^{\left(2\omega \right)}\;={\overset{↔}{\chi }}_{s}^{\left(2\right)}∶E^{\left(\omega \right)}\left(r\right)E^{\left(\omega \right)}\left(r\right)\delta \left(r-a\right)+{\overset{↔}{\chi }}_{b}^{\left(2\right)}:E^{\left(\omega \right)}\left(r\right)\nabla E^{\left(\omega \right)}\left(r\right),$$

(10) $${\overset{↔}{\chi }}_{s}^{\left(2\right)}=\chi_{s,⊥⊥⊥}^{\left(2\omega \right)}\vec{r}\vec{r}\vec{r}+\chi_{s,⊥∥∥}^{\left(2\omega \right)}\vec{r}\left(\vec{\theta }\vec{\theta }+\vec{\varphi }\vec{\varphi }\right)+\chi_{s,∥⊥∥}^{\left(2\omega \right)}\left(\vec{\theta }\vec{r}\vec{\theta }+\vec{\varphi }\vec{r}\vec{\varphi }+\vec{\theta }\vec{\theta }\vec{r}+\vec{\varphi }\vec{\varphi }\vec{r}\right),$$

(11) $$P_{bulk}^{\left(2\omega \right)}=\beta E_{in}^{\left(\omega \right)}\left(\nabla \cdot E_{in}^{\left(\omega \right)}\right)+\gamma \nabla \left(E_{in}^{\left(\omega \right)}\cdot E_{in}^{\left(\omega \right)}\right)+\delta^{\prime }\left(E_{in}^{\left(\omega \right)}\cdot \nabla \right)E_{in}^{\left(\omega \right)}$$

In the above equation, ${\overset{↔}{\chi }}_{s}^{\left(2\right)}$ and ${\overset{↔}{\chi }}_{b}^{\left(2\right)}$ represent the second-order surface polarizability and the bulk polarizability, respectively. ***E****(ω)* is the electric field vector at the fundamental wave and *δ* is the selection function that defines the surface of the OH-SiNB. $\chi_{s,⊥⊥⊥}^{\left(2\omega \right)}$, $\chi_{s,⊥∥∥}^{\left(2\omega \right)}$ and $\chi_{s,∥⊥∥}^{\left(2\omega \right)}$ are the surface nonlinear polarizability component, ⊥ and ‖ correspond to the components of the perpendicular and parallel surfaces. The *β*, *γ*, and *δ′* are the bulk polarizability component. Since the divergence of the electric field is zero in a homogeneous medium, the *β* term disappears. According to [41], at *λ* = 800 nm, we take $\chi_{s,⊥⊥⊥}^{\left(2\omega \right)}\;$= 65 × 10^−19^ m^2^/V, $\chi_{s,⊥∥∥}^{\left(2\omega \right)}$ = 3.5 × 10^−19^ m^2^/V. $\chi_{s,∥⊥∥}^{\left(2\omega \right)}$, *γ*, *δ′*, are 1 × 10^−19^ m^2^/V. We performed the numerical simulations of the SHG with COMSOL Multiphysics, following the methods described in references [31,42,43]. Our numerical simulation method supports the simulation results in [33,44] well.

**Figure 5:**
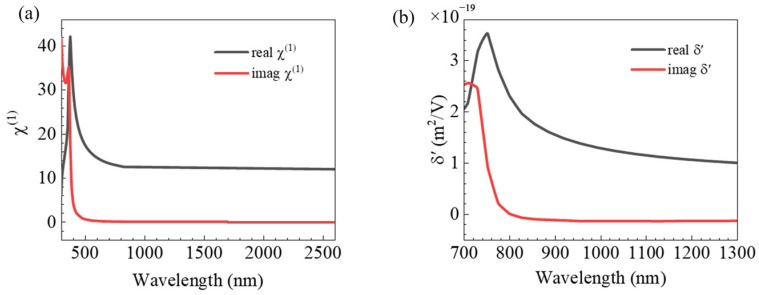
(**a**) Frequency dispersion of linear susceptibility of silicon. (**b**) Frequency dispersion of second-order susceptibility of silicon.

Because the second-order polarizability dispersion relation of silicon has not been well established, we use Miller’s law [45] to express the full-wave nonlinear polarizabilities. The relation is as in Equation (12).

(12) $$\frac{\chi^{\left(2\right)}\left(\Omega ,\omega \right)}{\chi^{\left(1\right)}\left(\Omega \right){\left[\chi^{\left(1\right)}\left(\Omega ,\omega \right)\right]}^{2}}=C$$

where *χ*^(2)^(Ω,ω) and χ^(1)^(Ω) are the nonlinear and linear polarizabilities, respectively, and *C* is a constant. Hence, second-order nonlinear polarizability *χ*^(2)^ at any frequency can be calculated when the constant *C* is known at a particular wavelength. We used the second-order nonlinear polarizabilities of silicon at *λ* = 800 nm to obtain the dispersion functions of $\chi_{s,⊥⊥⊥}^{\left(2\omega \right)}$, $\chi_{s,⊥∥∥}^{\left(2\omega \right)}$, $\chi_{s,∥⊥∥}^{\left(2\omega \right)}$, *γ* and *δ′*, as shown in Figure 5. It should be noted that because the dispersion curves of the other second-order nonlinear polarizabilities of the silicon only differ in amplitude, in Figure 5 we only show the second-order nonlinear polarizability of *δ′*.

**Figure 6:**
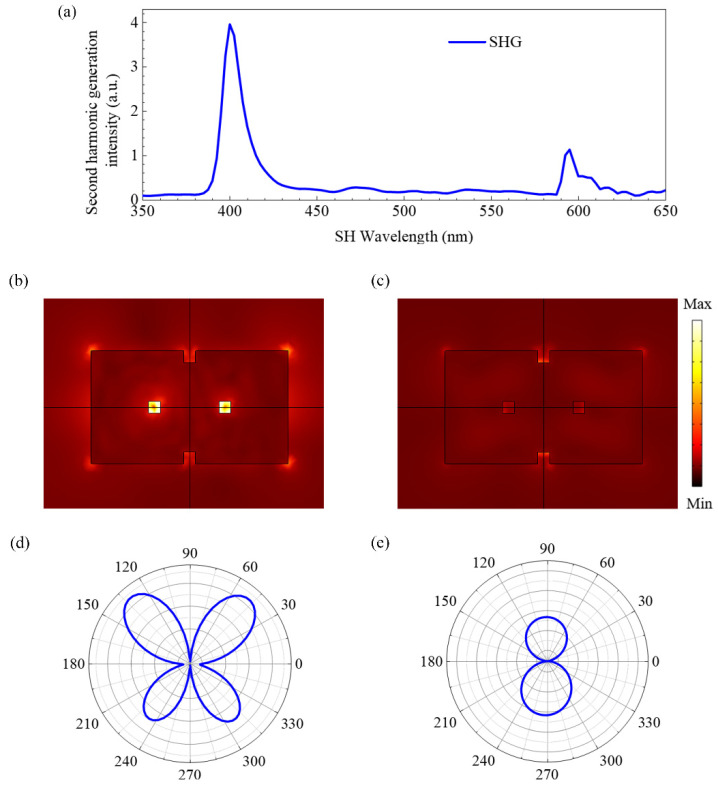
(**a**) The SHG intensity of the OH-SiNB. Near-field electric field distribution of SHG in the *xoz* plane at (**b**) *λ_SHG_*_1_ = 400 nm and (**c**) *λ_SHG_*_2_ = 595 nm. Angular plots of the SH far-field radiation in the *yoz* plane at (**d**) *λ*_SHG1_ = 400 nm and (**e**) *λ*_SHG2_ = 595 nm.

With the above dispersion relation for second-order polarizabilities, the SHG of the OH-SiNB is shown in Figure 6. Of concern are the two peaks of the forward scattering spectra (*λ*_SHG1_ = 400 nm and *λ*_SHG2_ = 595 nm), i.e., the relationship between the fundamental wavelengths (*λ*_1_ = 800 nm and *λ*_2_ = 1190 nm) and SHG. The peak of the fundamental field in Figure 2a agrees well with the peak of SHG shown in Figure 6a, which indicates that the electromagnetic field enhancement at the fundamental wavelength plays an important role in the enhancement effect of the SHG. The intensity of the SHG at *λ*_SHG1_ is about four times stronger than that at *λ*_SHG2_. The main reason for the stronger SHG intensity at *λ*_SHG1_ can be attributed to the surface electromagnetic field localization. The fundamental electromagnetic field distribution (Figure 4) shows that field enhancement is significantly stronger at *λ*_1_ = 800 nm than at *λ*_2_ = 1190 nm. This proves that the novel magnetic dipole mode influenced by the Fabry–Perot mode is effective in realizing the surface localization of the electromagnetic field and obtains strong effective coupling between light and matter. Another reason is that each second-order polarizability component at *λ*_1_ = 800 nm is higher than that at *λ*_2_ = 1190 nm. As shown in Figure 5, the second-order polarizability of silicon in our investigated band decreased with increasing wavelength. In addition, the intensity and efficiency of SHG enhancement were also affected by the spatial overlap ratio of the fundamental and second harmonic [46]. In Figure 6b,c, the near-field electric field distributions of SHG in the *xoz* plane at *λ_SHG_*_1_ = 400 nm and *λ_SHG_*_2_ = 595 nm are as shown. We observed significant SHG in the holes. This behavior also indicates the effectiveness of the holes. The far-field SH responses of the OH-SiNB are shown in Figure 6d,e. Different far-field modes can be observed by the excitation of incident light of different frequencies and polarization directions. For incident light polarized along the *x*-axis direction, the four-lobe pattern and a two-lobe pattern were observed in the *yoz* plane at *λ*_SHG1_ = 400 nm and *λ*_SHG2_ = 595 nm, respectively. It is noted that the far-field radiation-direction angles of *λ*_SHG1_ and *λ*_SHG2_ SH radiation correspond to different radiation patterns, especially at *λ*_SHG2_, which has a significant radiation asymmetry. As the small nanoparticles, the SH far-field radiation modes correspond to different higher-order multipole resonances. The SHG radiation pattern can be controlled by a fourth-order response, while for larger nanostructures with delay effects, it is necessary that octupole terms or higher-order terms should be considered [47]. The SHG radiation patterns are always closely related to multipole mode emissions [48]. Thus, there are some differences in the radiation patterns at dual wavelengths.

## 4. Conclusions

In summary, we demonstrated the directional forward-scattering enhancement and SHG enhancement at dual wavelengths in an OH-SiNB nanostructure by numerical simulations. We show the TED, MD, EQ, MQ and MD_1_ resonance modes as well as their interactions at dual wavelength by exploiting electromagnetic multipole decomposition. At *λ*_1_ = 800 nm, the directional forward-scattering enhancement effect is produced by the interaction of the MD_1_ and TED modes with the EQ mode, where the MD_1_ mode is a novel magnetic dipole influenced by the Fabry–Perot mode. The interaction of these three poles satisfies the generalized Kerker condition. At *λ*_2_ = 1190 nm, the conventional MD and TED mode interference, which approximately satisfies the first Kerker condition, results in near-zero backscattering. Comparing the radiation-direction angles at *λ*_1_ and *λ*_2_ in the *xoz* plane, there is a smaller directional angle of radiation at *λ*_1_ because of the EQ mode. The surface electromagnetic field of the square holes is significantly enhanced attributing to the effects of the Fabry–Perot mode and the magnetic dipole toroidal displacement current. The strong electromagnetic field located on the surface of the square holes and the increased surface area of the square holes efficiently achieved SHG enhancement at dual wavelength. The demonstrated SHG intensity at *λ*_1_ was four times higher than at *λ*_2_. The results show that our method provides a new strategy for designing nanoantennas and applying centrosymmetric materials to second-order nonlinear optoelectronic devices.

## Data Availability

Not applicable.
